# Femtosecond Laser‐Induced Recrystallized Nanotexturing for Identity Document Security With Physical Unclonable Functions

**DOI:** 10.1002/advs.202411449

**Published:** 2024-11-11

**Authors:** Panpan Niu, Jiao Geng, Qilin Jiang, Yangyundou Wang, Jianxin Sang, Zhenghong Wang, Liping Shi

**Affiliations:** ^1^ Hangzhou Institute of Technology Xidian University Hangzhou 311231 China; ^2^ School of Optoelectronic Engineering Xidian University Xi'an 710126 China; ^3^ Shanghai Guanzhong Optical Technology Co., Ltd. Shanghai 201900 China

**Keywords:** anti‐counterfeiting, femtosecond laser, nanotexture, physical unclonable functions, silicon

## Abstract

Counterfeit identity (ID) documents pose a serious threat to personal credit and national security. As a promising candidate, optical physical unclonable functions (PUFs) offer a robust defense mechanism against counterfeits. Despite the innovations in chemically synthesized PUFs, challenges persist, including harmful chemical treatments, low yields, and incompatibility of reaction conditions with the ID document materials. More notably, surface relief nanostructures for PUFs, such as wrinkles, are still at risk of being replicated through scanning lithography or nanoimprint. Here, a femtosecond laser‐induced recrystallized silicon nanotexture is reported as latent PUF nanofingerprint for document anti‐counterfeiting. With femtosecond laser irradiation, nanotextures spontaneously emerge within 100 ms of exposure. By introducing a low‐absorption metal layer, surface plasmon polariton waves are excited on the silicon‐metal multilayer nanofilms with long‐range boosting, ensuring the uniqueness and non‐replicability of the final nanotextures. Furthermore, the femtosecond laser induces a phase transition in the latent nanotexture from amorphous to polycrystalline state, rather than creating replicable relief wrinkles. The random nanotextures are easily identifiable through optical microscopy and Raman imaging, yet they remain undetectable by surface characterization methods such as scanning electron and atomic force microscopies. This property significantly hinders counterfeiting efforts, as it prevents the precise replication of these nanostructures.

## Graphical abstract

A femtosecond laser‐induced recrystallized nanotexturing technique is developed to create physical unclonable functions on multilayer nanofilms. By enhancing long‐range surface plasmon polaritons, the photochemical process generates unique recrystallized nanotextures within 100 milliseconds. These recrystallized nanotextures are easily identifiable through optical microscopy, yet they remain undetectable by other surface characterization methods. This feature significantly hinders counterfeiting by scanning and imprinting.

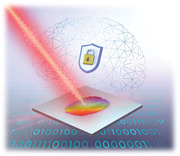

## 1 Introduction

Throughout the evolution of human civilization, the perennial confrontation between counterfeiting methods and anti‐counterfeiting strategies has continued unabated.^[^ ^1^ ^]^ Commercial counterfeits not only infringe consumer interests but also destabilize the market order and stifle technological innovation.^[^ ^2^ ^]^ The counterfeiting of high‐value identity (ID) documents such as licenses, passports, and ID cards presents a serious risk to national security,^[^ ^3^ ^]^ thereby necessitating relentless innovation in anti‐counterfeiting technology to maintain an edge in this ongoing confrontation. Currently, the predominant approaches involve the development of diverse anti‐counterfeiting tags using unique materials and intricate encoding to enhance the complexity of forgery. Nevertheless, due to the determinacy and predictability of material properties and encoding schemes, conventional anti‐counterfeiting tags can still ultimately be duplicated or tampered with.^1^ Highly unpredictable stochastic processes can thwart decryption effectively, prompting the emergence of physical unclonable functions (PUFs).^[^ ^4^, ^5^, ^6^ ^]^ The PUFs exploit the inherent randomness during the manufacturing processes of anti‐counterfeiting tags, they exhibit distinctive physical security that are inimitable, ensuring that even identical manufacturing processes cannot reproduce them.^[^ ^7^ ^]^

Nowadays, there already exist several anti‐counterfeiting strategies employing optical PUFs,^[^ ^8^, ^9^ ^]^ among which surface textures stand out as effective anti‐counterfeiting tags.^[^ ^10^, ^11^, ^12^ ^]^ These optical texturing PUF tags based on different techniques, e.g., high‐temperature annealing,^[^ ^13^ ^]^ wet spin coating,^[^ ^14^ ^]^ and chemical vapor deposition,^[^ ^15^ ^]^ etc. have garnered significant interest owing to their diverse random mechanisms, adjustable level of randomness, and broad applicability.^[^ ^16^, ^17^ ^]^ Notably, optical texturing PUF tags are appropriate for safeguarding paper or polymer ID documents that cannot be implanted with non‐flexible electronic anti‐counterfeiting chips.^[^ ^18^ ^]^ Surface disordered micro or even nano structures have been deSSSmonstrated to construct PUF anti‐counterfeiting textures, with the most typical structures being the chaotic wrinkling formed by internal stresses in polymers^[^ ^19^, ^20^ ^]^ or composite films.^[^ ^21^ ^]^ The recently developed PUF anti‐counterfeiting textures by stochastic chemical syntheses,^[^ ^22^ ^]^ such as stochastic dispersion of luminescent nanoparticles^[^ ^15^ ^]^ and photopolymerization of anisotropic liquid crystals,^[^ ^23^, ^24^ ^]^ further demonstrate its advantages in optical multi‐dimensional uniqueness.^[^ ^25^, ^26^, ^27^ ^]^ These miniature textures that rely on chemical materials and reactions provide high encoding capacity at limited physical scales through various optical reading and decoding methods, including visual microscope,^[^ ^21^ ^]^ Raman spectroscopy,^[^ ^28^ ^]^ fluorescence,^[^ ^29^ ^]^ and so on.^[^ ^25^ ^]^

For further stable deployment, optical PUF tags require robust physical patterns over a long period of time. Permanently inscribing the robust PUF patterns onto protected objects can effectively prevent tags from transferring and tampering. However, most patterned PUF tags are synthesized in wet‐chemical^[^ ^30^, ^31^ ^]^ or high‐temperature environments,^[^ ^13^, ^15^ ^]^ which may be incompatible with sensitive polymers. For instance, an increasing number of countries are adopting more environmentally friendly polycarbonate (PC) polymer materials for their latest ID cards and passport security pages, but currently, neither PC cards nor films acquire texturing PUF tags through chemical synthesis directly, due to their hydrolytic aging in high temperature and humidity environments.^[^ ^32^ ^]^ Moreover, the instability of chemically synthesized textures, with short lifespan of fluorescent substances, cumbersome manufactures and identification processes also limits its application in ID document anti‐counterfeiting.^[^ ^19^ ^]^

Unlike nanostructures by slow chemical syntheses, pulsed laser‐induced surface structures^[^ ^33^ ^]^ represent a universal phenomenon capable of generating scalable nanoripples on diverse materials including metals, semiconductors, dielectrics, and polymers in terms of the interference between incident laser and launched surface electromagnetic waves.^[^ ^34^, ^35^ ^]^ These structures have enhanced stability and durability, their effectiveness in optics, mechanics, and biochemistry has been substantiated.^[^ ^36^ ^]^ However, during the formation of nanoripples, surface electromagnetic waves are strongly perturbed by the scattering of the original as well as laser‐induced rough surfaces, causing the distortion and intersection of nanoripples, particularly under static spot irradiation.^[^ ^37^, ^38^ ^]^ Due to this stubborn problem, the irregularity of nanoripples has always been considered as a disadvantage for its widespread applications. As a result, previous investigations into laser‐induced ripples have primarily focused on achieving highly regular nanostructures to facilitate specific surface functionalization.^[^ ^39^, ^40^, ^41^ ^]^

In this work, we demonstrate that one can take advantage of the intrinsic disorder of recrystallized silicon (Si) nanotexturing to fabricate PUF anti‐counterfeiting tags, which can be rapidly generated on ID documents through femtosecond laser exposure within 100 milliseconds. As precursors of nanotextures, Ti‐Cu‐Si multilayer nanofilms were deposited on polymer ID card through physical vapor deposition (PVD) at room temperature. By adding metal layers to the nanofilm, surface plasmon polariton (SPP) waves are excited with long‐range boosting, inducing the disordered texturing of the recrystallized Si through femtosecond laser irradiation. These 2D nanotextures of recrystallized Si can serve as latent nanofingerprints with ability to resist scanning and imprinting duplicate. Furthermore, the spatially shaping and homogenizing of femtosecond laser based on orthogonal polarization tailoring can print identification blocks as small as 50 µm×50 µm, with an encoding capacity of 10^170^ for PUF construction. In the realm of applied concepts, a neural network featured by its lightweight framework and swift operation, is employed to demonstrate an artificial intelligence identification strategy, which includes 1000 true anti‐counterfeiting tags along with 200 fake ones for test. Compared to electronic and chemical PUF tags, the femtosecond laser‐induced recrystallized Si nanotexturing tag with simplicity, flexibility, and compatibility, while taking into account the aesthetics of the large‐area patterns with dynamic structural colors, shed new light on the application of femtosecond laser to optical PUF anti‐counterfeiting for ID document security.

## 2 Results

### 2.1 Femtosecond Laser‐Induced Nanotextures on Silicon‐Metal Nanofilms

**Figure 1:**
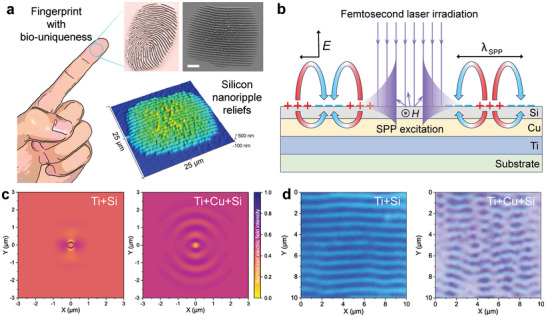
Femtosecond laser‐induced silicon nanotextures for PUF. a) Fingerprint inspired motivation for femtosecond laser‐induced Si nanotextures. Grayscale morphology and 3D morphology of the femtosecond laser‐induced Si nanoripples, were characterized by scanning electron microscope (SEM) and atomic force microscope (AFM), respectively. The Si nanoripples possess a regular and surface relief nanostructure, making them easily replicable by nanoimprinting. b) Schematic illustration of nanotextures formation induced by femtosecond laser irradiation. The imperfect surface of the Si film scatters the laser exposure and excites surface plasmon polaritons (SPPs). c) Simulated electric field distributions on laser‐illuminated Ti‐Si and Ti‐Cu‐Si films. The Cu film enhances the surface electric field intensity and interference between the SPP source through long‐range SPP boosting. d) Optical microscope images of femtosecond laser‐induced nanotextures on Ti‐Si and Ti‐Cu‐Si films with Si substrates. Scale bar: 5 µm (a).

Biological fingerprints possess undeniable bio‐uniqueness and are widely utilized in authentication.^[^ ^42^ ^]^ Fingerprints contain distinct line patterns discernible to the naked eye, these patterns are ridges and grooves that can be accurately deciphered for security identification through optical detection methods. Beyond this, as a fingerprint uniquely grow on an individual finger, their use in authorization builds a direct link between ID information with a specific person. By introducing artificial nanoscale fingerprints, ID documents can be protected against counterfeiting and unauthorized access. Such fingerprint inspired PUF can be skillfully realized by the unique nanoripples on single layer Si film (**Figure** **1**a), which directly grow on the material surfaces through easily implementable femtosecond pulsed laser illumination.^[^ ^43^ ^]^

However, the laser‐induced nanoripples exhibit a self‐organizing effect, resulting in orderly arrangements. Significantly, these nanoripples are surface reliefs, like biological fingerprints, carry the risk of being replicated through scanning and molding, especially with the advancement of nanoimprint technology. In the general electromagnetic model underlying the self‐organization of nanotextures on Si film,^[^ ^44^ ^]^ the irradiation of linearly polarized femtosecond laser onto the Si surface results in the scattering of incident light due to surface roughness. This process is accompanied by the Si absorption of partial light energy, which subsequently excites SPPs that propagate along the film surface. The interference of these SPP waves with the incident light further leads to spatial modulation of the energy distribution on the Si surface, culminating in the formation of a ripple‐like texture through spatially selective reaction. As a consequence, the key to disrupting regular ripples lies in enhancing the propagation of SPP waves in various directions along the Si surface, as well as in the random coupling between SPP waves and incident laser energy. In addition, during the pulsed laser irradiation, the absorption and scattering, as seeds of the nanotextures, both trigger oxidation and phase transition of Si. The heterogeneity of the irradiated Si, characterized by variations in its absorption properties and surface roughness, further introduces an inherent randomness to the nanotexturing process, ultimately resulting in the creation of nanotextures that are random and lack a straight arrangement.

For a start, in order to improve the adhesion between Si nanofilms and polymer substrates of ID documents, Ti metal is used as an adhesive layer^[^ ^45^ ^]^ for the deposition of amorphous Si. However, in the formation process of oxidative Si nanotextures, the electromagnetic near‐field effect is also an important factor.^[^ ^46^ ^]^ The Ti metal layer exhibits a high absorption of femtosecond laser energy, which enhances the near‐field effect and subsequently limits the propagation of SPP waves. Consequently, the femtosecond laser‐induced nanotexture exhibit a regular, strip‐like grating structure, lacking inherent randomness and morphological features, which plays a pivotal role in defining their utility in PUF. To disrupt the regular self‐organizing ripples on the Si film, a Cu metal layer with relatively low absorption is introduced below the Si layer (Figure 1b). This Cu layer serves to isolate the Si layer from the highly absorbent Ti layer while maintaining the adhesion of the multilayer nanofilm. More importantly, the Cu layer introduces additional long‐range SPP boosting,^[^ ^46^ ^]^ further enhancing the surface electric field intensity and the SPP interference, resulting in more disordered nanotextures rather than regular ripples. A schematic simulation demonstrates this enhancement, Figure 1c shows the simulated electric field intensity distributions on laser‐irradiated Ti‐Si and Ti‐Cu‐Si films. Taken overall, the surface electric field intensity of Ti‐Cu‐Si is higher than that of Ti‐Si, and the excited SPP waves have longer propagation, which leads to the far‐field effect that determines the disordered nanotexture.

By optimizing the thickness of the multilayer film (as detailed in Note S1, Supporting Information), the Ti, Cu, and Si layers are chosen with thicknesses of 100 nm, 100 nm, and 50 nm, respectively, to facilitate the generation of standardized nanotextures. By irradiating Ti‐Si and Ti‐Cu‐Si films on a Si substrate with a 370 fs laser, the nanotexture of the multilayer nanofilm with a Cu layer exhibit disordered meandering and branching, while the film without Cu layer remains relatively regular (Figure 1d). The Cu layer not only introduces randomness to the nanotexture but also possesses better thermal conductivity than the Ti layer,^[^ ^47^ ^]^ allowing the laser‐induced Si nanotextures to be directly generated on most ID documents that utilize polymer materials. High single‐pulse energy femtosecond laser acting on multilayer nanofilm generates thermal accumulation, even if the exposure time is very short. Due to the low thermal conductivity of Ti metal, the multilayer nanofilm with a single Ti layer on polymer substrate is susceptible to damage from thermal energy back impact^[^ ^48^ ^]^ and high thermal expansion. The Ti‐Cu‐Si film on the polymer PC substrate maintains a random nanotexture (Figure S1a, Supporting Information), while the Ti‐Si film is severely cracked. Because the seed effect of these cracks,^[^ ^49^ ^]^ only some regular ripples are generated along the cracks (Figure S1b, Supporting Information). The laser energy threshold for thermal accumulation damage is significantly lower than that for random nanotexture formation, which cannot be mitigated by reducing the irradiation flux. Therefore, the incorporation of a Cu layer presents a dual advantage for femtosecond laser generated nanotextures directly on polymer substrates.

Moreover, the nanotexture formation on Si‐metal multilayer nanofilm is acutely sensitive to laser energy, even slight variations in exposure time and flux can unpredictably affect nanotextures. Consequently, the structural morphology of nanotextures eludes precise prediction through self‐organizing models reliant on diffusion, dynamics, or erosion processes.^[^ ^37^ ^]^

### 2.2 Spatially Shaped Femtosecond Laser for Anti‐Counterfeiting Tags Printing

**Figure 2:**
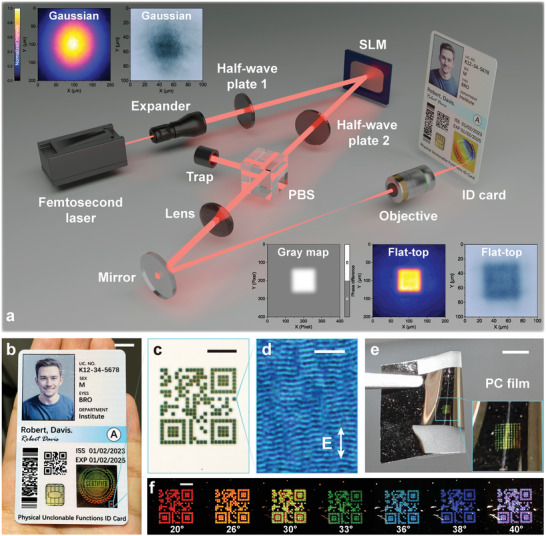
PUF nanotexture printing by spatially shaped femtosecond laser for ID document security. a) Schematic of the femtosecond laser spatial shaping system based on orthogonal polarization tailoring. The original non‐uniform Gaussian spot is shaped into a flat‐top block to achieve uniform nanotexture printing. b) Simulated ID card with PUF anti‐counterfeiting tag by spatially shaped femtosecond laser printing. c) Micro‐QR code on the certified tag constructed from PUF nanotexture blocks, which encodes the word “Hello”. d) Optical microscope image of the PUF nanotexture within a printing block. e) Femtosecond laser printing block matrix on a flexible PC film. f) Dynamic structural colors of the PUF micro‐QR code from different illumination angles (the observation angle is fixed at 10° and the illumination angle increases from 20° to 40° approximately), illustrating the optical diffraction of nanotextures. Scale bars: 10 mm (b), 500 µm (c, f), 5 µm (d) and 5 mm (e).

As a surface nanotexture, the Si nanofilm as precursor is sensitive to laser flux. The nanotexture produced by a single Gaussian spot is uneven, corresponding to the Gaussian energy distribution (**Figure** **2**a). This unevenness hinders the exploration of nanotexture characteristics for PUFs and the printing of visual anti‐counterfeiting patterns for application. Therefore, spatial shaping of the initial femtosecond laser light field is beneficial for improving the quality of the nanostructures.

Figure 2a describes the femtosecond laser spatial shaping system based on orthogonal polarization tailoring for nanotexture printing. The femtosecond laser with vertical polarization is expanded and incident onto a reflective spatial light modulator (SLM) through the half‐wave plate 1. A grayscale map with a gradient‐edged block is loaded onto the SLM reflection surface,^[^ ^50^ ^]^ creating a π phase difference between the block area and the part intended to be filtered out. Subsequently, through the combination of the half‐wave plate 2 and a polarizing beam splitter (PBS), the block and other area within a Gaussian spot are spatially separated due to the orthogonal polarization caused by the π phase difference. To maintain the pattern of the block and reduce the beam size, a 4*f* system is employed, and finally, the block‐shaped femtosecond laser is focused onto the surface of the multilayer nanofilm through an objective. Using the spatially shaped femtosecond laser for anti‐counterfeiting tag printing can make nanotextures more effective for PUFs, with a more uniform distribution of energy density within a flat‐top light field. The spatial shaping of femtosecond laser based on orthogonal polarization tailoring has the flexibility to achieve printing of irregular micropatterns while maintaining high‐quality nanotextures (Figure S2, Supporting Information). This capability is valuable for anti‐counterfeiting applications, providing a faster and more cost‐effective alternative to photolithography.

The design of the anti‐counterfeiting tag features a pixel matrix composed of skillfully arranged nanotexture blocks. A polyvinyl chloride simulated ID card with PUF anti‐counterfeiting tag by spatially shaped femtosecond laser printing is shown in Figure 2b. Alongside macroscopic patterns, tiny blocks enable the printing of microscopic patterns, facilitating the incorporation of miniature anti‐counterfeiting applications. On the certified pattern side, a micro‐QR code constructed from PUF nanotexture blocks encodes the word “Hello” (Figure 2c). Within these blocks, the flat‐top femtosecond laser induces rich nanotextures (Figure 2d), conferring the visual patterns with unclonable features. It is worth noting that, as a low‐spatial‐frequency laser‐induced periodic structures,^[^ ^37^ ^]^ the mainstream orientation of nanotexture propagation is perpendicular to the laser polarization. Figure 2e shows nanotexture block matrix on a flexible PC film. Owing to the cold processing characteristics of pulsed femtosecond lasers, nanotexture blocks can be directly printed on flexible thin film substrates. This advancement further extends the application of laser‐induced nanotextures to flexible paper documents, such as passport pages.

In contrast to other optical PUF tags that require special excitations, it is noteworthy that the dynamic structural colors of tags are observable by the naked eye under the visible light. Within individual identification blocks, the nanotextures on the film surface have a relatively regular alignment, which can be regarded as nanogratings for optical diffraction. The varying structural colors dependent on the angles of the illumination light and the observation (Figure S3, Supporting Information), are still feasible throughout the visible part of the spectrum^[^ ^51^ ^]^ (as shown in Figure 2f and Video S1, Supporting Information), even though these random nanotexture do not have a strict‐matching spatial period. While optical variable devices with regular gratings are common for anti‐counterfeiting, they offer limited security in level 1.^[^ ^52^ ^]^ In contrast, these durable nanotextures furnish steadfast structural colors and unclonable random nanostructures with higher level security, facilitating inkless color printing and effective anti‐counterfeiting functions for security tags.

### 2.3 Femtosecond Laser‐Induced Recrystallization of Amorphous Silicon

The femtosecond laser induces random nanotextures on the Si nanofilm, which are clearly observed using an optical microscope. However, once these textures form surface relief structures, they risk being replicated through nanoimprinting. Unlike wrinkles produced by internal stress of materials, the latent morphology and recrystallization of femtosecond laser‐induced Si nanotextures are difficult to be duplicated precisely.

**Figure 3:**
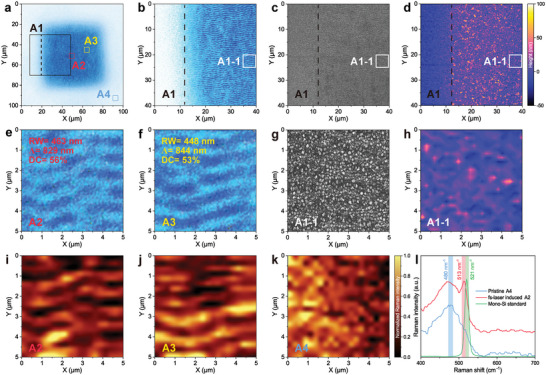
Femtosecond laser‐induced recrystallization of amorphous silicon. a) Optical microscope images of a femtosecond laser printing block. b–d) Optical microscope, SEM, and AFM images of the boundary area A1 in a the block. e, f) Enlarged views of the nanotexture areas A2 and A3 in a the block (RW, ridge width; Λ, period; DC, duty cycle). g, h) Enlarged views of nanotexture area A1‐1 in c, d A1. i–k) Raman mapping images of nanotexture areas A1, A2, and pristine area A4. l Raman spectra of pristine area A4, femtosecond laser induced A1, and mono‐Si standard.

We characterized the nanotextures within an identification block using optical microscope (**Figure** **3**a), scanning electron microscope (SEM), atomic force microscope (AFM), and Raman spectroscopy microscopy. In boundary area A1, optical microscopy reveals clear random nanotextures within the block (Figure 3b), while no distinct nanotextures are observed outside the block. The observable nanotextures display periodic ripples with a ridge width of RW = ≈450 nm, a period of Λ = ≈830 nm, and a duty cycle (ridge width/period) of DC = ≈55%. These dimensions align with the features of laser‐induced surface periodic structures at a wavelength of 1030 nm.^[^ ^37^ ^]^ However, surface morphology analyses of area A1 by SEM (Figure 3c) and AFM (Figure 3d) lack clear textures, only the boundary is visible in the AFM image.

The femtosecond laser‐induced nanotextures are generated in an ambient air environment, which naturally incorporates an oxidation reaction. Consequently, oxygen atoms are injected into the nanotexture areas, causing an obvious increase in the oxygen content in the Si film. To quantify this, we employed energy‐dispersive X‐ray spectroscopy (EDS) to analyze the oxygen contents in both the nanotexture and their surrounding areas. The EDS mapping (Figure S4, Supporting Information) and spectra (Figure S5, Supporting Information) demonstrated an obvious rise in oxygen content within the nanotexture areas, increasing by 69 wt% compared to the pristine areas (Table S1, Supporting Information). This significant increase indicates the formation of silicon oxide, driven by the photochemical reaction under the femtosecond laser irradiation. The femtosecond laser induces nanotextures on the surface of Si nanofilms (Figure 3e,f), while also producing additional transparent oxide silica attached on the block (Figure 3g,h). This oxide layer shields the underlying disordered nanotextures, which exhibits a characteristic rough particle morphology at the microscopic scale. Owing to the relatively uniform oxidation induced by spatial femtosecond laser, the grain size varies between 100 nm and 200 nm (Figure S6, Supporting Information). Additionally, the nanotextures with this protective oxide layer have a feature height of 19 nm approximately (Figure S7, Supporting Information). As a result, these nanotextures can be observed with an optical microscope but cannot be accurately measured by surface characterization techniques. That is, the nanotextures cannot be replicated by scanning or imprinting either. Raman mapping images of the nanotexture areas A2 and A3 (Figure 3i,j) reveal corresponding ripple textures observed in the optical microscope images (Figure 3e,f), whereas the blank area A4 (Figure 3k) does not exhibit any distinct textures.

Figure 3l shows the measured Raman spectra of areas A4, A2, and standard monocrystalline Si. The original Si nanofilm by PVD exhibits amorphous with a peak at 480 cm⁻¹, while monocrystalline Si shows the characteristic peaks at 521 cm⁻¹ as expected. After irradiation by the femtosecond laser to produce nanotextures, an additional peak appears at approximately 513 cm⁻¹, indicating that the femtosecond laser simultaneously induces the transformation of Si from amorphous to polycrystalline state. Accurately replicating the latent nanotexture covered under a sturdy oxide layer, with crystal state changes of Si is a challenge, and therefore the Raman mapping images can serve as a level 3 security of anti‐counterfeiting feature for PUFs. Simultaneously, the characterization results from SEM and AFM also indicate that the recrystallized Si nanotextures induced by femtosecond laser possess the ability to resist scanning and imprinting replication, laying the foundation for the development of advanced PUFs.

### 2.4 Morphology of the PUF Nanotexture Blocks

**Figure 4:**
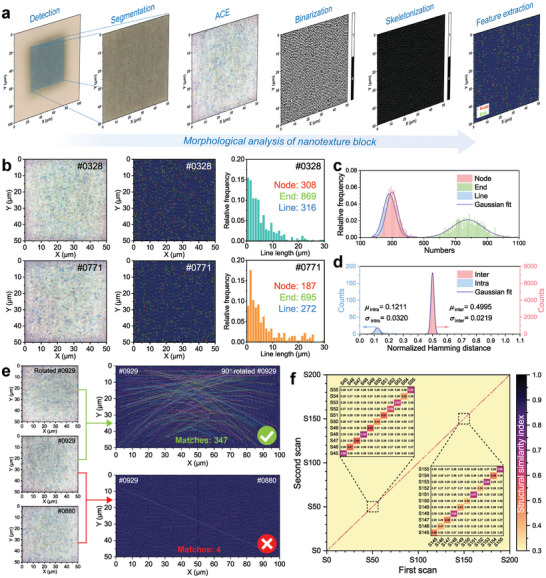
Morphology of the PUF nanotexture blocks. a) Flow chart of the digital image processing for morphological analysis of nanotexture blocks. First, the nanotexture area was segmented from the original block image. Second, the nanotexture within was prominent through automatic color enhancement (ACE). Thirdly, use edge detection algorithm to binarize the nanotexture. Next, the skeleton was characterized based on binarized nanotexture. Finally, features were extracted from the skeleton. b) Morphological comparison of two nanotexture blocks. Two different blocks have random nanotextures with different skeletal nodes, ends, and lines. c) Frequency distributions of skeletal node, end, line numbers of 1000 nanotextures. The numbers of nodes, ends and lines satisfy normal distribution with Gaussian fit, indicating the randomness of the skeletal features. d) Normalized Hamming distances of 200 intra‐blocks and 39 800 inter‐blocks. e) Skeletal match of the same and different blocks through algorithm matches. (e) Heatmap of matches between two scanning skeletons derived from 200 blocks. Each block underwent two scans, and the analysis was conducted between these image pairs. The color bar indicates the normalized structural similarity, values along the diagonal correspond to identical blocks, while off‐diagonal values represent comparisons between different blocks.

Utilizing consistent induction parameters, a set of 1000 PUF blocks was printed onto a Ti‐Cu‐Si film with a PC substrate. Their microscopic images were subsequently captured for morphological analysis via digital image processing, as illustrated in **Figure** **4**a. Images of individual blocks with a size of 1024 pixels×1024 pixels were segmented from the original images, which were then accentuated in their nanotexture using an automatic color enhancement (ACE) algorithm.^[^ ^53^ ^]^ Through a binarization process informed by edge detection, the nanotextures were abstracted as white, revealing the recrystallized ripples within. Viewed holistically from a single nanotexture, these striated ripples arranged in proximity, each maintaining a roughly uniform width yet differing in curvature, thereby displaying a distinctive natural dynamism and fluidity. Within these ripples, various distinct regions can be discerned, such as bifurcations (referred to as node points) and brief interruptions (referred to as end points), which highlight the sophistication of recrystallized nanotextures. Encouragingly, the natural sophistication of these latent 2D nanotextures render them challenging to replicate or imitate, thus bolstering the security from level 1 to level 3 in the development of optically variable PUF anti‐counterfeiting tags.

Further refinement in extracting the skeleton of the nanotextures yields a more distinct revelation of their structural features and growth trajectories. Due to the flat‐top light field generated by spatial beam shaping, the uniform femtosecond laser flux ensures complete filling and homogeneous distribution of nanoripples within the block. Figure 4b shows a morphological comparison of two nanotexture blocks (numbers #0328 and #0771 in Source Data file). Different nanotexture blocks possess distinct skeletal features, with varying node, end, and line numbers, as well as distinct 2D spatial distributions. Such features are particularly important for the PUF, guaranteeing the uniqueness of each block. Numbers of nodes, ends, lines for 1000 block skeletons undergo statistical analysis, as depicted in Figure 4c. The nanotexture blocks formed under a fix induction parameter, exhibit a distorted skeleton structure, with the relative frequencies of node, end, and line numbers following a random process by Gaussian fitted normal distribution. Additionally, the normalized Hamming distances of 200 intra‐blocks and 39800 inter‐blocks are concentrated at 0.1211 and 0.4995 (Figure 4d), respectively, with ideal values being 0 and 0.5. This further indicates the uniqueness of the femtosecond laser‐induced nanotextures.

To compare the uniqueness level of PUF blocks, we used the scale‐invariant feature transform algorithm^[^ ^54^ ^]^ to match the skeletons of nanotextures. Even if the image is rotated, the detected matches of the same nanotexture skeleton (number #0929) are significantly more than that of different one (number #0880), and these nanotextures are difficult to distinguish with the naked eye as shown in Figure 4e. In brief, fewer matches indicated lower similarity and higher uniqueness of nanotextures. As expected, the heatmap based on structural similarity index^[^ ^55^ ^]^ of two scans for the same set of nanotexture block skeletons presents a diagonal pattern (Figure 4f), underscoring the uniqueness and reliability of identification blocks with various PUF nanotextures.

Despite their random feature, disordered nanotextures are tightly packed within a confined area, offering an encoding capability of up to 10 ^170^ for a 50 µm×50 µm identification block (Note S2, Supporting Information),^[^ ^56^ ^]^ which surpasses the capacity threshold of 10^20^ for unclonable security.^[^ ^22^ ^]^ Not only that, anti‐counterfeiting tags employing nanotextures can be readily decoded using standard optical microscopes, diminishing the reliance on complex imaging systems while maintaining robust security. This approach enables the potential integration of a comprehensive authentication system into a portable mobile device.

### 2.5 Authentication of the Nanotexture PUF Tags

Optical PUF offers anti‐counterfeiting textures characterized by unique encoding, thereby enabling seamless reading and decoding through image identification algorithms. However, conventional image matching algorithms are limited by the high identification latency due to heavy feature extraction and individual comparison of numerous PUF images. To address this, we have demonstrated a lightweight artificial intelligence neural network strategy with low latency for nanotexture PUF.

**Figure 5:**
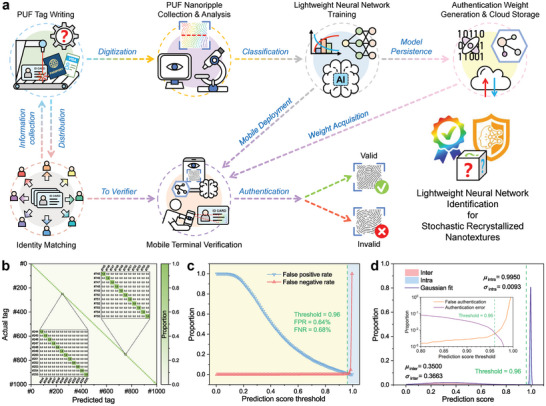
Lightweight neural network identification of PUF nanotexture tags. a) conceptual strategy of nanotexture PUF with a lightweight neural network identification for ID document security. b) Confusion matrix heatmap of trained MobileViT for 1000 tags classifications. The color bar represents the proportion of actual tags classified by MobileViT as predicted tags. The deep color diagonal signifies the correct predictions of actual tags aligning with predicted tags, demonstrating the accurate identification capability of MobileViT. c) Proportion of false positive rate and false negative rate for 12800 batch identification under a varying prediction score threshold. When the threshold is 0.96, the false positive rate (FPR) and the false negative rate (FNR) are 0.64% and 0.68%, respectively. d) Prediction score distributions of 6400 true and 6400 false tags. Inset: cumulative distribution functions indicating the probabilities of the false authentication (FA) and authentication error (AE) as a function of decision threshold for MobileViT identification.

**Figure** **5**a illustrates the conceptual strategy of nanotexture PUF with a lightweight neural network identification for ID document security. To begin with, nanotexture PUF security tags are directly inscribed onto ID documents, with each tags acquiring uniqueness owing to the random process deviations in manufacturing. The images of identification nanotexture blocks hidden in these visual tags are then captured and analyzed in batches, generating datasets for subsequent neural network training. Afterwards, the datasets comprising nanotexture images linked to ID matching, are fed into a lightweight neural network, which classifies the nanotexture images and generates identification weights for model persistence. Different from heavy neural network models, lightweight neural networks offer the advantage of deployment on mobile devices. This enables end‐users to efficiently utilize them for identification with smartphones, simply by accessing identification weights stored in the cloud. Consequently, efficient anti‐counterfeiting strategies of lightweight neural networks can contribute to PUF applications based on encoded nanotextures.

To evaluate our envisaged identification strategy, we constructed a neural network containing 1000 individual anti‐counterfeiting tag (using an identification block as a tag,) and executed identification tests. The lightweight MobileViT model,^[^ ^57^ ^]^ characterized as a general‐purpose, low‐latency, and mobile‐friendly vision transformer, is ideal for mobile visual classification tasks. MobileViT, as a ready‐to‐use black box, facilitate the rapid establishment of a workflow to validate the anti‐counterfeiting capability of nanotextures. Hence, MobileViT was selected as the basic framework for nanotexture PUF identification. In a standard identification workflow, nanotexture images acquired via microscopic imaging devices are directly fed into the MobileViT as identification tokens. Subsequently, a normalized prediction score of classification probability is generated, and the authenticity of an unidentified document can be adjudicated based on a predefined threshold (Note S3, Supporting Information). For nanotexture images, MobileViT is replaceable, given that the optical PUFs based on digital image recognition provide elevated adaptability and compatibility in the selection of identification algorithms.

Considering the differences in readout environments and devices of the end‐users, the datasets for MobileViT (64000 images in total) are composed of the original microscopic images of 1000 nanotexture blocks and their augmentations (Note S4, Supporting Information). Through multi‐angle rotations, brightness adjustments, color temperature changes, and the additions of noises to the original and ACE images, these augmentations simulate the nanotexture images captured under various conditions, thereby improving the robustness and generalization ability of identification network. As training epochs progressed, the train loss of MobileViT steadily declined, the validation accuracy improved, and both eventually converge (Figure S8, Supporting Information). After training for 300 epochs, the resulting confusion matrix distinctly demonstrated the high classification accuracy of trained MobileViT (Figure 5b), with Top‐1 and Top‐5 accuracy reaching up to 98.53% and 99.64%, respectively.

To further test the security of identification network, we challenged the trained MobileViT with a batch of 12800 images consisting of 200 true (re‐augmented 6400 images) and 200 false (re‐augmented 6400 images) tags, then output the prediction scores through the normalized probability function of neural network. According to the fact that MobileViT determines the authenticity of tags by prediction probabilities, the false positive and false negative rates under different thresholds of prediction score are revealed (Figure 5c). As the threshold of prediction score elevates below 0.96, there is a corresponding decrease in the false positive rate (FPR), whilst the false negative rate (FNR) remains unchanged. In order to achieve high safety capabilities, the false negative rate can be slightly higher than the false positive rate. Therefore, the threshold can be set to 0.96, and the FPR and the FNR are 0.64% and 0.68%, respectively. Furthermore, the regenerated 6400 images of true tags and the 6400 images of false tags are defined as the “intra‐tags” and the “inter‐tags”,^[^ ^58^ ^]^ respectively. The prediction score distributions of true and false tags are calculated as Figure 5d, the false authentication (FA) and authentication error (AE) rates according to the cumulative distribution functions are shown in the inset of Figure 5d. It can be seen that the true tags and the false tags are separated in score distributions, the prediction scores of true tags closed to 1.0 are significantly higher than that of false tags, owing to the pre‐training characteristics of neural network. The decision threshold of 0.96 corresponds to estimated FA and AE rates of 0.60% and 0.69%, respectively. It should be noted that the identification of unidentified documents should not rely on single identification block, and multi‐block identification can be realized by leveraging the abundant nanotextures contained in the anti‐counterfeiting tag patterns, thereby further reducing FA and AE. Meanwhile, the identification speed of MobileViT was assessed by the time consumption of batch identifying the 12800 images, and the average identification speed is 2885 second/12800 tags = 0.23 second/tag.

MobileViT leverages the spatial inductive biases of convolutional neural networks and the global representation learning capacity of vision transformers, which is particularly crucial for recognizing PUF nanotexture patterns effectively. Utilizing the fast and accurate recognition of MobileViT, multiple identification blocks can be hidden within a single PUF tag, achieving a higher security PUF strategy. As an optional identification method, MobileViT demonstrates the effectiveness of femtosecond laser‐induced nanotextures as robust PUF entities for identity document security, highlighting not only their inherent security benefits but also the superior disorder entropy source of the nanotextures. This aligns with the results of morphological analysis of the nanotextures, further emphasizing their practical applications in the real world.

## 3 Discussions

In our work, we have proposed a nanotexturing PUF tag composed of recrystallized Si blocks for ID document security. The nanotextures with random nanoripples are rapidly induced by ultrafast femtosecond laser irradiation on a Ti‐Cu‐Si multilayer nanofilm. The inherent randomness of nanoripples generated by long‐range SPP boosting, establishes the foundational basis for the unpredictability of PUF, whereas their nanograting structure contributes to the stable structural color essential for patterned tags. The spatial shaping of femtosecond laser spot, based on orthogonal polarization tailoring, provides a uniform and flexible light field for the generation of high‐quality nanotextures and graphic printing. Surface characterization and morphological analysis of these nanotextures further acknowledge that the 2D disordered recrystallized Si nanoripples can be utilized as latent nanofingerprints for PUF implementation. The construction and assessment of a lightweight neural network‐based identification strategy, covering 1000 tags, have substantiated the promising potential of nanotextures in ID verification and anti‐counterfeiting applications.

In contrast to other PUFs synthesized through slow chemical processes, femtosecond laser‐induced nanotextures on the material surface represent a more convenient and rapid PUF generation approach. A single 50 µm×50 µm nanotexture block, endowed competitive encoding capacity of 10 ^170^ and feature density of ≈10 ^168^ µm^−2^, can be generated in a mere 100 milliseconds for mass production. Although our design does not yet match the encoding capabilities of PUFs that utilize multidimensional spectral encoding methods (Table S2, Supporting Information), it aims to offer an efficient anti‐counterfeiting strategy with a streamlined manufacturing and reading process, particularly well‐suited for applications in identity document security. While many femtosecond laser patterning methods focus on surface texturing, our approach introduces femtosecond laser‐induced recrystallization as a critical mechanism. Unlike conventional methods that primarily create regular patterns or reliefs,^[^ ^59^, ^60^ ^]^ our technique induces a phase transition in the Si nanolayer from amorphous to polycrystalline states, resulting in highly random and non‐replicable nanotextures with transparent oxide shields. This recrystallization process is a novel feature that significantly enhances the PUF properties of the nanotextures, making them unique and inherently resistant to replication. The selective recrystallization process of nanoripples operates independently of unstable chemical procedures, avoiding expensive lithography, harmful chemical treatments, and inflexible pattern masks. The resulting non‐photolithographic, self‐organized nanotextures are scalable, tunable, and cost‐effective, mitigating the trade‐off between customization and production efficiency in large‐area anti‐counterfeiting tags associated with the patterned mask method for ID document security.

Patterned femtosecond laser‐induced nanotexturing PUF tags establish a functional and aesthetically appealing identification framework by leveraging visual structural color spectrum, stochastic nanotextures derived from standard optical images, and corresponding Raman mapping with multi‐level security (Table S3, Supporting Information). This identification methodology proves both convenient and efficient, facilitating seamless integration into mobile device identification systems. Nevertheless, the intrinsic limitations of optical microscopic images hinder their ability to fully exploit the Raman spectrum information of recrystallized Si nanoripples, thereby constraining the encoding capacity of nanotexture PUF. Significantly, the pursuit of enhancing femtosecond laser‐induced nanotextures through the exploration of multidimensional spectral encoding techniques, such as photoluminescence and Raman scattering, stands as a promising avenue to elevate the security level of PUF.^[^ ^61^, ^62^ ^]^ It is acknowledged, however, that this advancement may entail an increased workload on readout devices.

In summary, the innovative use of femtosecond laser‐induced nanotextures in developing optical PUF anti‐counterfeiting tags offers foreseeable advantages. First of all, replicating the irregular latent 2D nanotextures is exceedingly challenging,^[^ ^37^ ^]^ rendering it highly secure as an anti‐counterfeiting pattern. Secondly, femtosecond laser induces a spatial distribution of recrystallization corresponding to the morphology of nanotextures, endowing the recrystallized Si nanotextures with the potential to develop extensible anti‐counterfeiting capabilities through multi‐dimensional spectra. Finally, the capability of femtosecond laser to modulate the surface, encompassing marking, decoration, data storage, encryption, and anti‐counterfeiting, appropriately aligns with the requirements for ID document security in terms of security, aesthetics, and efficiency.

The femtosecond laser‐induced recrystallized Si nanotextures, acting as artificial nanofingerprints, inherently possess anti‐replicating/tampering qualities and uniqueness suitable for PUFs, promising versatile applications in anti‐counterfeiting across various domains, from commercial products to ID document security. We hope that the proposed femtosecond laser‐induced recrystallized Si nanotextures can further expand the applications of femtosecond lasers and serve as inspiration for applying more interesting microscopic phenomena with randomness to PUF development.

## 4 Experimental Section

### Simulations

The simulated electric field distributions on laser‐illuminated Ti‐Si and Ti‐Cu‐Si multilayer nanofilms were performed by using Ansys Lumerical FDTD software. In the simulations, scattered‐field source at 1030 nm wavelength was launched to 100 nm Ti‐50 nm Si and 100 nm Ti‐100 nm Cu‐50 nm Si film model with a constant scattering source and SiO_2_ substrate. Scattering source was simplified by adding a SiO_2_ waveguide with size of 200 nm×200 nm×100 nm in the Si layer. The models were divided into 15 nm×15 nm×2 nm meshes for electric field intensity calculation.

### Materials

Sputtering targets of Ti (99.995%), Cu (99.995%), and Si (99.999%, P‐type) for magnetron sputtering were provided from Beijing Zhongnuo Advanced Material Technology Co., Ltd. Polyvinyl chloride, polycarbonate cards and films were provided from Shanghai Guanzhong Optical Technology Co., Ltd.

### Manufacturing of the Nanotexture PUF Tags

The 100 nm Ti‐50 nm Si and 100 nm Ti‐100 nm Cu‐50 nm Si multilayer nanofilms were deposited on polyvinyl chloride, polycarbonate cards and films by a high‐vacuum magnetron sputtering system (SKY Tech, PVD500) at 25 °C, DC power of 100 W, and Ar flow of 30 sccm.

A commercial femtosecond laser (ULTRA, OR‐20‐IR) served as laser source, providing pulse width of 370 fs at a central wavelength of 1030 nm. The pulse repetition rate of laser was kept at 10 kHz in all experiments for block printing. The exposure time and flux of laser were 100 ms and 0.125 J cm^−2^, respectively. The spatial phase delay of femtosecond laser light field was modulated by a reflection‐type liquid crystal spatial light modulator (UPOLabs, HDSLM80R Plus, resolution of 1920×1200, single pixel width of 8 µm, 10 bit of 1024 grayscale levels, fill factor 95%). The 4*f* system consists of a single convex lens (*f*_1_ = 600 mm) and a long working distance NIR objective (×5/0.15, *f*_2_ = 40 mm). Patterned laser printing was achieved through a 3‐axis motorized stage (Lianyi, XM118+ZM100).

### Characterizations

The optical microscopic bright‐field images of the nanotextures for morphological analysis and neural network identification were captured by an optical metallurgical microscope (SOPTOP, RX50M) equipped with objective (SOPTOP, LMPlanFL 20×/0.40, 50×/0.75, 100×/0.85). SEM images of nanoripples and nanotextures were obtained by scanning electron microscopy (ZEISS, GeminiSEM 450 and HITACHI, SU8600) combined with EDS detector (Oxford Instruments, Ultim Extreme) for mapping and spectra analysis. Topographic AFM images of the nanotextures were measured by atomic force microscope (Oxford Instruments, Jupiter XR). Raman microscopy was analyzed by confocal Raman imaging microscope (WITec, Alpha 300R).

### Morphological Analysis of the Nanotexture Blocks

Morphological analysis was performed using the Python language in the PyCharm environment (Python 3.11+PyCharm 2023.2). The optical microscopic images of 1000 nanotexture blocks were processed with segmentation, automatic color enhancement (ACE), binarization, and skeletonization. Individual nanotextures were then subjected to skeletal feature extraction, including statistics on node, end, line numbers, and line length.

### Lightweight Neural Network Identification Strategy

The neural network identification strategy was built based on the original framework of MobileViT model without other pretrained weights. The training and classification of identification strategy was executed by the PyTorch library (2.0.1+cu118) with a CUDA device (NVIDIA GeForce RTX 4090).

The identification strategy used for demonstration contains 1000 true tags. The training and validation data sets were generated through a self‐written Python script. The original and ACE images of a single tags were used as source and augmented to a total of 64 different images, these 64 images were used to simulate images obtained under unideal conditions, such as unconventional lighting, pollution, damage, digital noise, through augmentations including different rotation angles, grayscale, brightness, saturation, color temperature, blur, masking, noise, etc. 64000 images generated from 1000 true tag images were divided into training data set (52000 images) and validation data set (12000 images) according to a ratio of 13:3 for each tag (52:12 images). A detailed augmentation information of tag images was given in Note S4 (Supporting Information). The original 1200 images (1000 true, 200 fake) and partial augmented image samples were provided in Source Data file.

The model of the identification strategy was trained for 300 epochs with a batch size of 96, each epoch required 4 min∼5 min, resulting in a total training time of about 13 hours–16 hours for 1000 true tags.

### Code Availability

The Python codes for morphological analysis and neural network identification provided together with the source data in the Source Data file.

## Conflict of Interest

The authors declare no conflict of interest.

## Supporting information



Supporting Information

Supplementary Video S1

## Data Availability

The data that support the findings of this study are openly available in DataSet for Femtosecond laser‐induced recrystallized nanotexturing for identity document security with physical unclonable functions at https://doi.org/10.6084/m9.figshare.26829061.v4, reference number 26829061.
