# Supplementary material for: Femtosecond Laser‐Induced Recrystallized Nanotexturing for Identity Document Security With Physical Unclonable Functions
Source: Adv Sci (Weinh). 2024 Nov 11;12(1):2411449. doi: 10.1002/advs.202411449 (PMC11714173; doi:10.1002/advs.202411449)
Supplement: Supplementary file 1 — Supporting Information [file ADVS-12-2411449-s002.docx]

Supplementary Information for

**Femtosecond laser-induced recrystallized nanotexturing for identity document security** **with physical unclonable functions**

Panpan Niu^1^, Jiao Geng^1,2^, Qilin Jiang^1^, Yangyundou Wang^1^, Jianxin Sang^3^, Zhenghong Wang^3^, Liping Shi^1,2,*^

^1^*Hangzhou Institute of Technology, Xidian University, Hangzhou 311231, China*

^2^*School of Optoelectronic Engineering, Xidian University, Xi’an 710126, China*

^3^*Shanghai Guanzhong Optical Technology Co., Ltd., Shanghai 201900, China*

**Corresponding author: shiliping@xidian.edu.cn*

**List of Contents**

Figure S1. Optical microscope images of femtosecond laser-induced nanotextures on Ti-Si and Ti-Cu-Si films with PC substrates 4

Figure S2. Femtosecond laser printing of the irregular micro patterns based on orthogonal polarization tailoring 5

Figure S3. SEM image of the nanoparticles of silicon oxide on the nanotextures 6

Figure S4. EDS mapping of the nanotexture for elemental composition analysis 7

Figure S6. SEM image of the nanoparticles of silicon oxide on the nanotextures 9

Figure S7. Edge profile of the identification block for feature height characterization by AFM 10

Figure S8. Train loss and validation accuracy of MobileViT with training epochs 11

Table S1. Analysis results of elemental composition 12

Table S2. A comparison between the femtosecond laser-induced nanotexture tag and the other optical PUF tags 13

Table S3. Laser-induced recrystallized nanotextures with multi-level security 14

Note S1. Optimization of Ti-Cu-Si multilayer nanofilm thicknesses 15

Note S2. Calculation of the encoding capacity 18

Note S3. Validation workflow of MobileViT 23

Note S4. Augmentation information of tag images 24

Legend of Video S1. Dynamic structural colors of the PUF micro-QR code 27

References 28


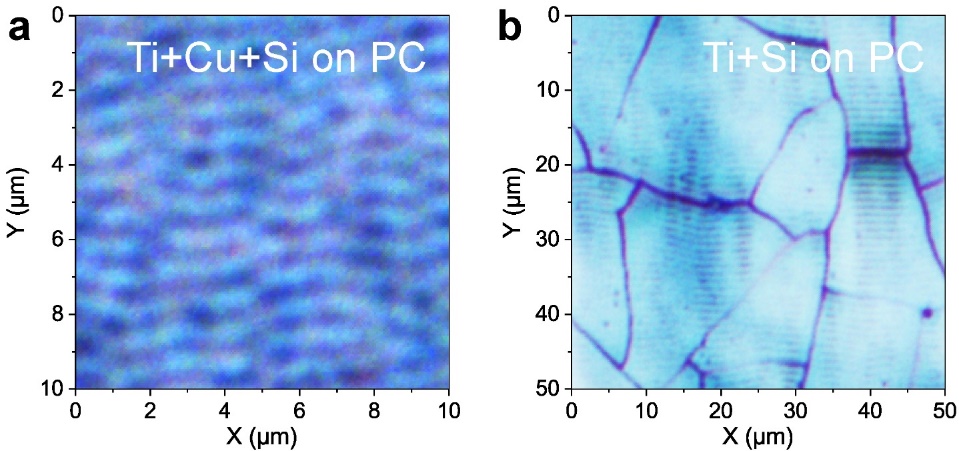


**Figure S1.** Optical microscope images of femtosecond laser-induced nanotextures on a Ti-Si and b Ti-Cu-Si films with PC substrates. The addition of a Cu layer induces disordered nanotextures and enhances thermal conductivity to prevent Si film cracking.


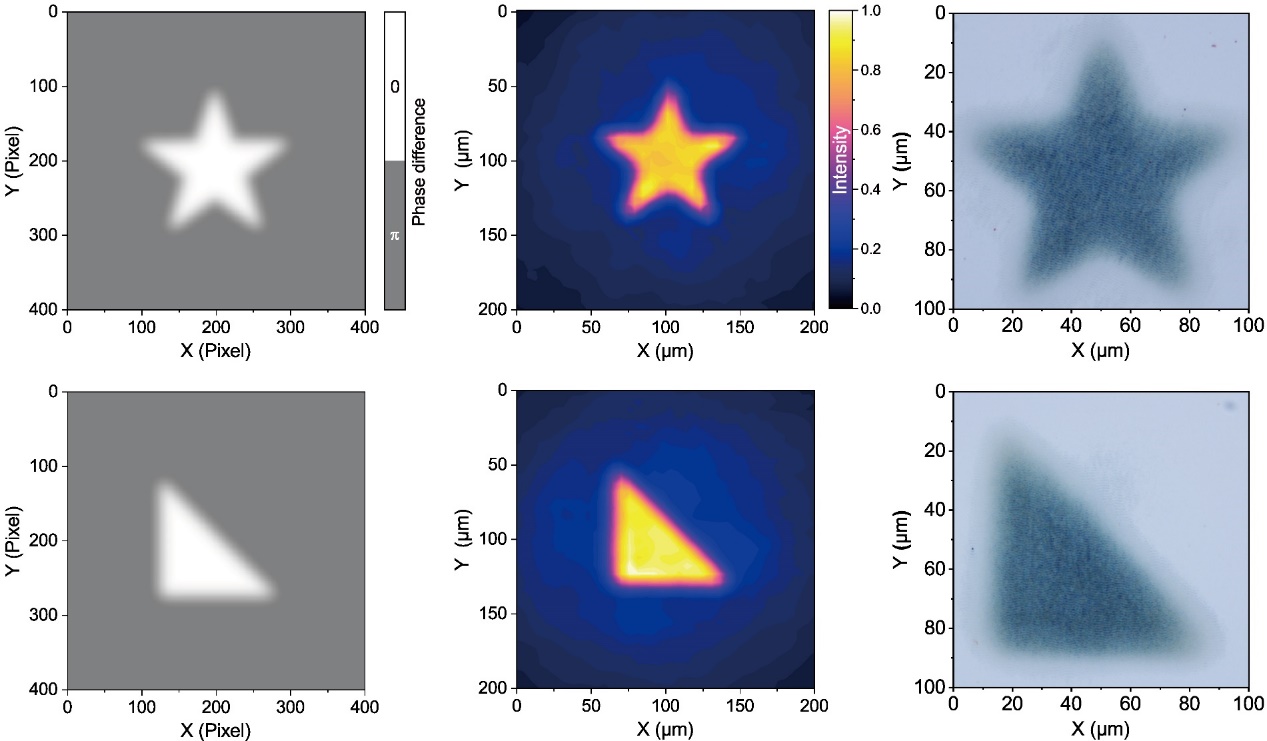


**Figure S2.** Femtosecond laser printing of the irregular micro patterns based on orthogonal polarization tailoring.


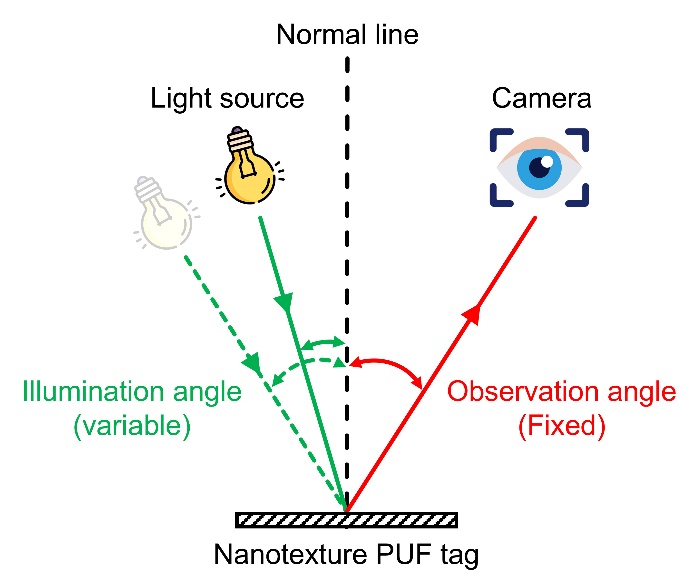


Figure S3. SEM image of the nanoparticles of silicon oxide on the nanotextures. The observation angle is fixed at 10° and the illumination angle increases from 20° to 40° in our experiments.


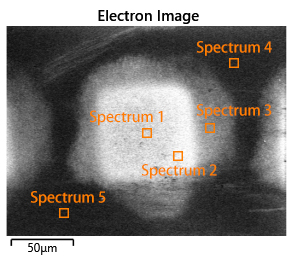

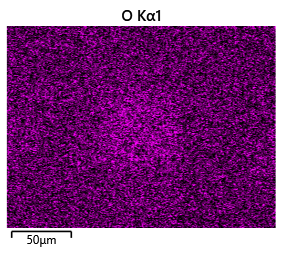


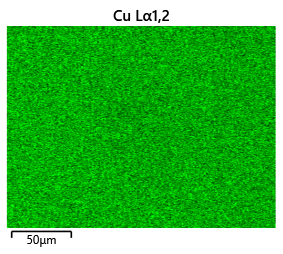

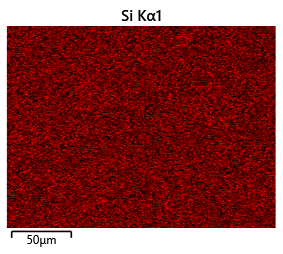


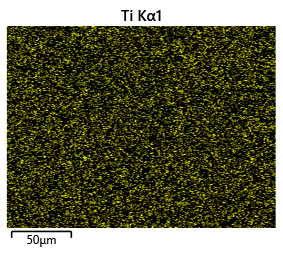

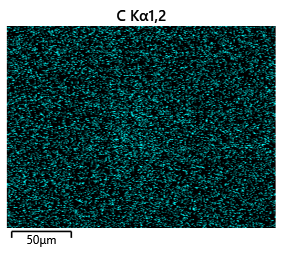


**Figure S4.** EDS mapping of the nanotexture for elemental composition analysis.


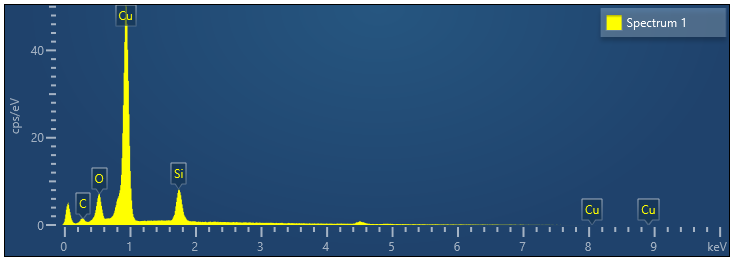


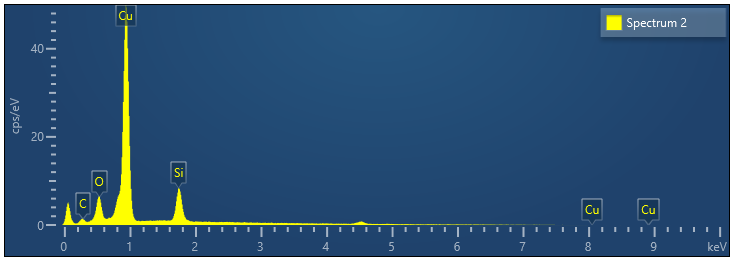


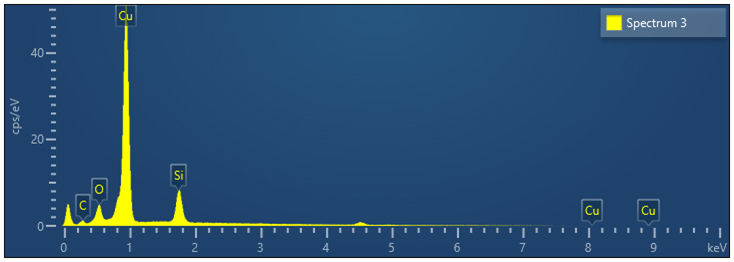


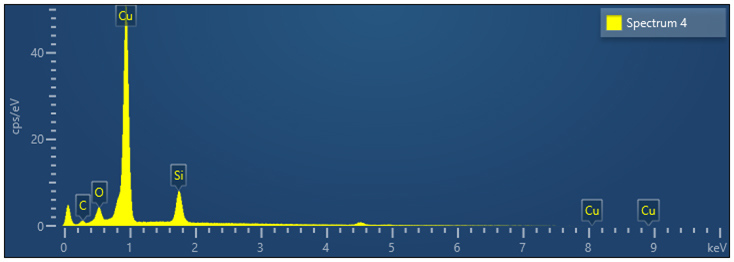


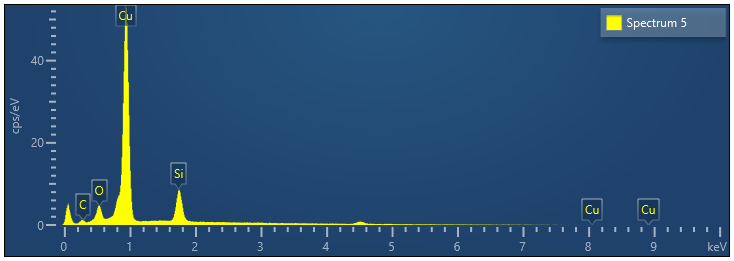


Figure S5. EDS spectra of the different areas corresponding to Figure S4.


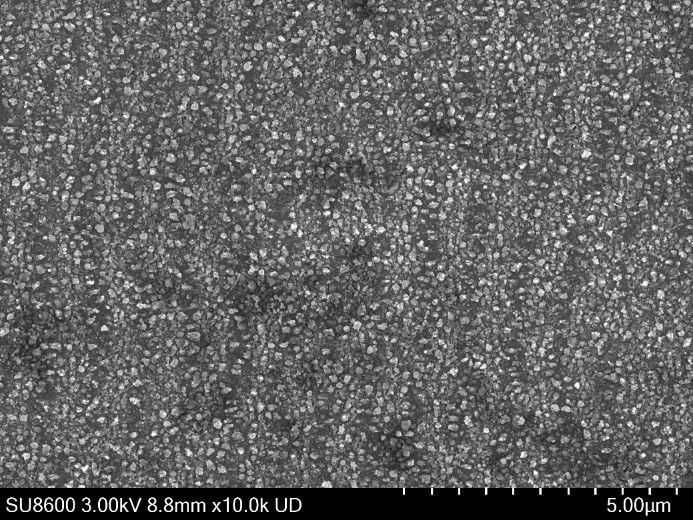


Figure S6. SEM image of the nanoparticles of silicon oxide on the nanotextures.


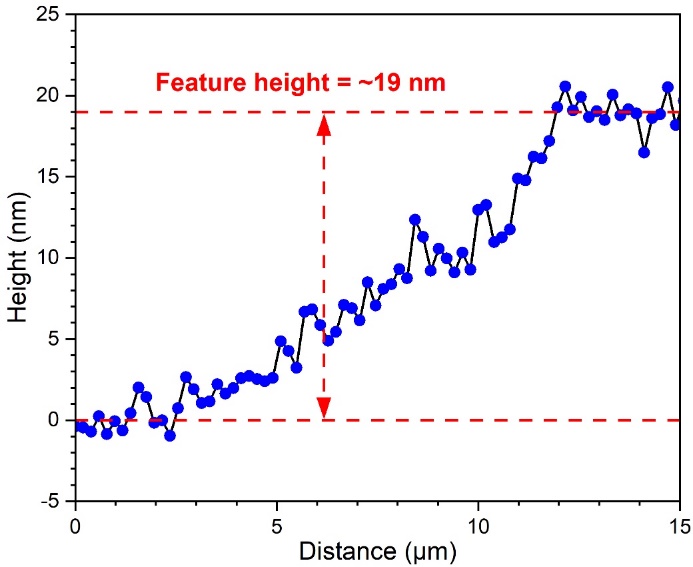


Figure S7. Edge profile of the identification block for feature height characterization by AFM.


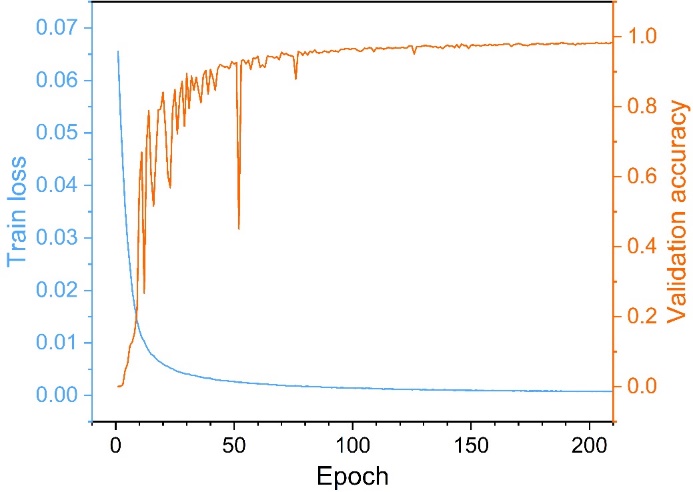


Figure S8. Train loss and validation accuracy of MobileViT with training epochs.

Table S1. Analysis results of elemental composition.

| Element | **Spectrum 1**  **(nanotexture)** | **Spectrum 2**  **(nanotexture)** | **Spectrum 3**  **(transition)** | **Spectrum 4**  **(pristine)** | **Spectrum 5**  **(pristine)** |
| --- | --- | --- | --- | --- | --- |
| C | 3.59 wt% | 3.32 wt% | 2.96 wt% | 2.64 wt% | 2.62 wt% |
| **O** | **6.40** wt% | **5.96** wt% | **4.05** wt% | **3.48** wt% | **3.83** wt% |
| Si | 10.60 wt% | 10.97 wt% | 11.15 wt% | 11.10 wt% | 11.08 wt% |
| Cu | 79.41 wt% | 79.74 wt% | 81.84 wt% | 82.78 wt% | 82.47 wt% |
| Total | 100.00 wt% | 100.00 wt% | 100.00 wt% | 100.00 wt% | 100.00 wt% |

Table S2. A comparison between the femtosecond laser-induced nanotexture tag and the other optical PUF tags.

| PUF strategy | Encoding capacity | Effective area  (μm^2^) | Feature density  (Encoding capacity/ Effective area, /μm^2^) | Readout method |
| --- | --- | --- | --- | --- |
| Rare-earth ion doped silica nanocomposite^4^ | 10^100^ | 100 | 10^98^ | Confocal laser scanning microscopy |
| Revisiting silk^5^ | 7.17×10^103^ | 10^6^ | ~10^98^ | Lens-free optical microscopy |
| Lanthanide doped polymer film^6^ | 6×10^104^ | 900 | ~10^102^ | Confocal laser scanning microscopy |
| Polymeric particle^7^ | 10^135^ | 7854 | ~10^131^ | Confocal laser scanning microscopy |
| Multilayer metal nano copolymer^8^ | 2.83×10^136^ | 785 | ~10^133^ | Raman microscopy |
| Aluminum oxide infiltrated polymer film^9^ | 10^169^ | 282,743 | ~10^164^ | Optical microscopy |
| Gap-enhanced Raman scattering nanoparticle^10^ | 6×10^15,051^ | 10,000 | ~10^15,048^ | Raman microscopy |
| Polymer carbon dots nanofilm^11^ | 10^63,593^ | 22,500 | ~10^63,589^ | Fluorescence scan and white-light interferometry |
| femtosecond laser-induced nanotextures (our work) | 10^170^ | 2500 | ~10^168^ | Optical microscopy |

Table S3. Laser-induced recrystallized nanotextures with multi-level security.

| Security level | Inspection method | Conventional anti-counterfeiting | Laser-induced attributes in our work |  |
| --- | --- | --- | --- | --- |
| Level 1 | Naked eye | Optically variable devices, color-shifting inks, etc. | Variable structural colors pattern by naked eye | 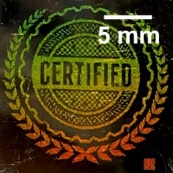 |
| Level 2 | Simple equipment | Microprinting, UV/IR inks, etc. | Random nanotextures by optical microscope | 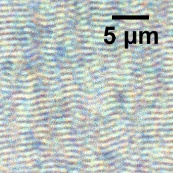 |
| Level 3 | Specialized equipment | Covert taggants, etc. | Recrystallized nanotextures by Raman spectroscopy | 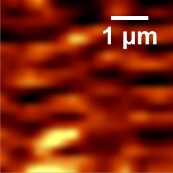 |

Note S1. Optimization of Ti-Cu-Si multilayer nanofilm thicknesses.

As a multilayer nanofilm, the thickness of each layer can potentially influence the final nanotexture produced. The Ti layer, serving as the adhesion layer for the substrate and other layers, has a minimal effect on the nanotexture, as it is isolated from the propagation of surface plasmon polaritons (SPPs) by the Cu layer. The Cu layer, with its lower absorption for femtosecond lasers, introduces long-range SPPs boosting, which are key to the formation of nanotextures. Therefore, we initially used the FDTD software to simulate the electric field distributions at the interface between the Si and Cu layers of the laser-illuminated Ti-Cu-Si films. The Ti layer is fixed at 100 nm and the Si layer at 50 nm, while the thickness of the Cu layer varied from 5 nm to 150 nm, as shown in Figure S9. The simulation results indicate that as the thickness of the Cu layer *t*_Cu_ increases, the electric field does not exhibit significant changes once the Cu thickness exceeds 20 nm. This is due to the limitation of skin depth at 20 nm~30 nm. Over the wavelength range of interest, it is possible to compute the skin depth via:

$\delta=\frac{1}{\mathrm{Re}\sqrt{-k_{0}^{2}\varepsilon_{r}}}$ (S1)

where *k*_0_ is the free space wave number, and *ε_r_* is the complex-valued relative permittivity. In conclusion, the preferred range for the thickness of the Cu layer is between 50 nm and 100 nm, which is sufficient to ensure the thermal protection capability on the polymer substrate.


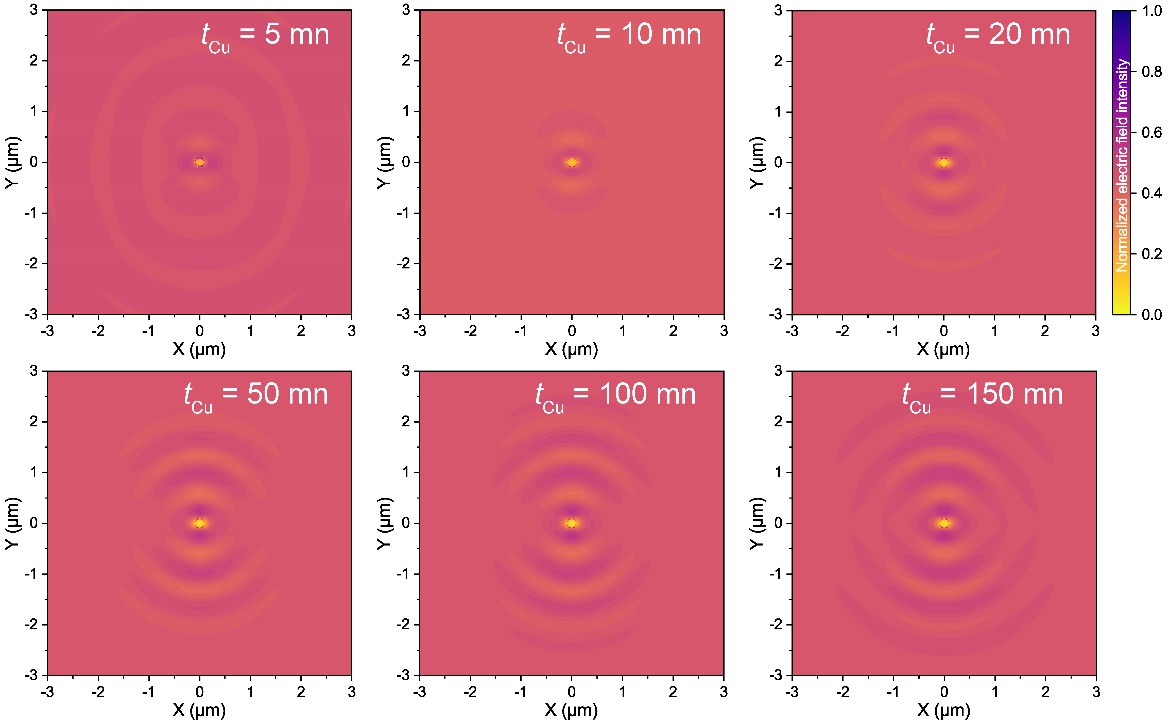


**Figure S9.** Simulated electric field distributions on laser-illuminated Cu-Si films with varying Cu layer thicknesses.

Based on our previous research^1-3^, the thickness of the top Si layer has a more significant influence on nanostructure formation than the Cu layer. We prepared Cu-Si nanofilms on Si substrates via magnetron sputtering, keeping the Cu thickness constant at 100 nm while varying the Si layer thickness *t*_Si_=15 nm~70 nm. Femtosecond laser pulses with a wavelength of 1030 nm were employed to induce nanostructures on the surface of nanofilms. The optical microscopy images of theirs nanotextures are shown in Figure S10. Due to the variation in thickness of the Si layer, the colors of the nanotextures appear different under the microscope. As the Si layer thickness increases, the femtosecond laser-induced nanostructures deviate from their initially ordered state, a change influenced by multiple factors. On the one hand, a thicker Si layer leads to a weakening of the SPP excitation with Cu layer, which in turn reduces the interference between the incident femtosecond laser and the SPPs, causing a gradual loss of control over the self-organized regularity. On the other hand, the thicker Si layer results in greater thermal accumulation, altering the absorption of free carriers in the Si layer. This can affect the efficiency of the laser-material interaction, and the rapid increase in temperature may lead to irregular melting and resolidification processes on the Si surface, thereby affecting the regularity of the periodic structures. But in reality, increasing the Si thickness elevates the threshold for laser induction, significantly slows the growth rate of the nanotextures. Furthermore, an increased thickness of the Si layer results in a higher production of silicon oxides, which imparts a diffuse brown hue. This coloration can obscure the nanotextures, complicating their identification. Consequently, these factors impede the suitability of nanofilms for the fabrication of PUFs. Thus, experimental results suggest the optimal thickness range of the Si layer is 30 nm~60 nm.

Additionally, for nanofilms fabricated using physical vapor deposition, sufficient thickness facilitates film formation and uniformity, as well as more precise thickness control. Therefore, in our experiments, the thicknesses of Ti, Cu, and Si are selected as 100 nm, 100 nm, and 50 nm, respectively, to generate standardized nanostructures.


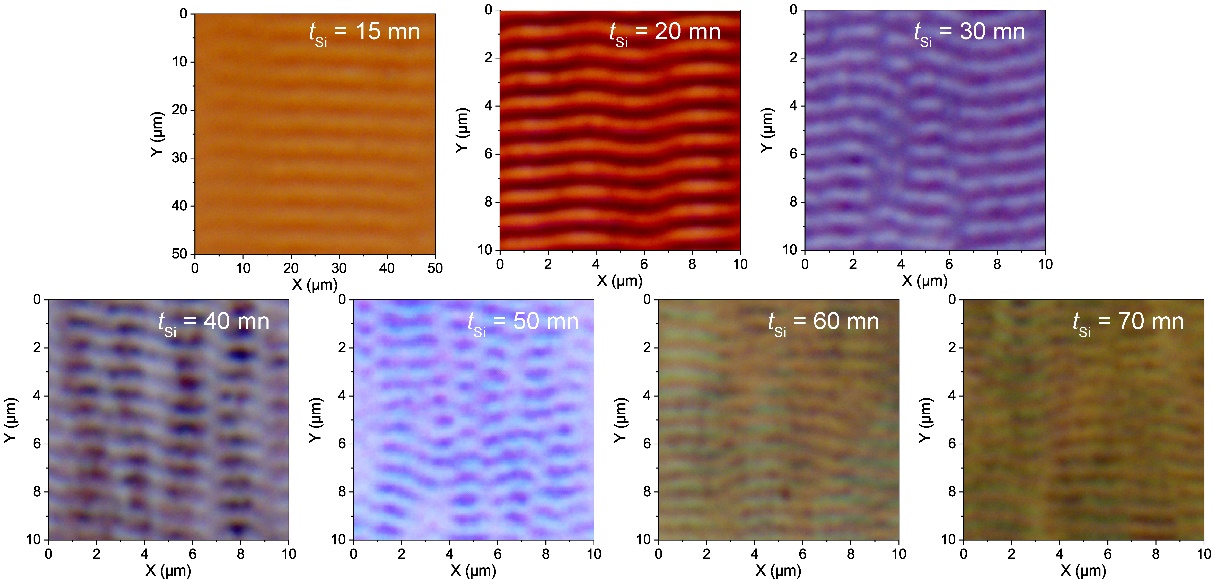


**Figure S10.** Optical microscopy images of the nanotextures on Cu-Si nanofilms with varying Si layer thicknesses. Due to the variation in thickness of the Si layer, the colors of the nanotextures appear different under the microscope.

Note S2. Calculation of the encoding capacity.

Encoding capacity is an important indicator for estimating PUFs, and a larger encoding capacity means that PUFs can be more difficult to crack. Currently, there are two popular methods for evaluating encoding capacity of PUFs. The first approach is predicated on the number of bits *C* that a single storage unit can encode and the total count of such units *m*. The encoding capacity is *C^m^*. In the context of patterned PUFs, *C* represents the color bit depth of an individual pixel, for instance, binary grayscale corresponds to *C*=2, while RGB color model corresponds to *C*=3. The variable *m* denotes the total number of pixels within the pattern image. However, this straightforward calculation tends to overestimate the actual encoding capacity, as it fails to account for the feature size present in the physical pattern. Consequently, a second method proposed by Carro-Temboury *et al*.^6^, which takes into consideration the physical dimensions of the pattern features, is deemed more appropriate for evaluating the encoding capacity of patterned optical PUFs. This method has been adopted in our work to provide a more accurate estimation. Specifically, the encoding capacity of nanotextures is determined through the skeleton analysis of computer vision, as described in the Morphology of the PUF nanotexture blocks section of the main text. These skeletons represent the critical feature pathways of nanotextures and serve as a vital foundation for distinguishing nanotexture PUFs. By leveraging the structural information provided by these skeletons, we can more accurately assess the encoding capacity and unique of nanotextures. For an accurate skeleton analysis, the optical microscopic images of nanotexture blocks are subjected to a computer vision processing flow that includes segmentation, automatic color enhancement, binarization, and skeletonization. In this processing flow, the binarization of nanotextures is a classic edge detection task for computer vision.

To be more specific, the edge detection of nanotextures is achieved through the convolution of the original nanotexture image with a kernel (operator), as shown in Figure S11. Both the image and the kernel can be regarded as two-dimensional matrices, where the kernel acts as a filter that is applied across the input image. To make it simple, the kernel move over the whole image, from left to right, from top to bottom by applying a convolution product. The output of this operation is a filtered grayscale image, which can be used for the next step of skeletonization after binary threshold judgment. Various operators can serve as the kernel, such as Sobel, Canny, and Laplacian operators. For our analysis, we have chosen the Laplacian operator (https://docs.opencv.org/4.x/d5/db5/tutorial_laplace_operator.html#autotoc_md689), a second-order derivative operator renowned for its use in image processing and edge detection. Laplacian operator identifies edges by calculating the second derivative of the image's pixels, making it well-suited for detecting areas of rapid intensity change within the image. The direction independence of Laplacian operator allows it to detect edges in all directions by assessing changes across the entire image landscape. It is clear that the matrix size of the operator plays a pivotal role in the outcomes of edge detection and subsequent skeleton analysis. Consequently, selecting an appropriately sized operator is crucial for ensuring that the skeleton extracted through computer vision analysis closely matches the actual nanotexture.


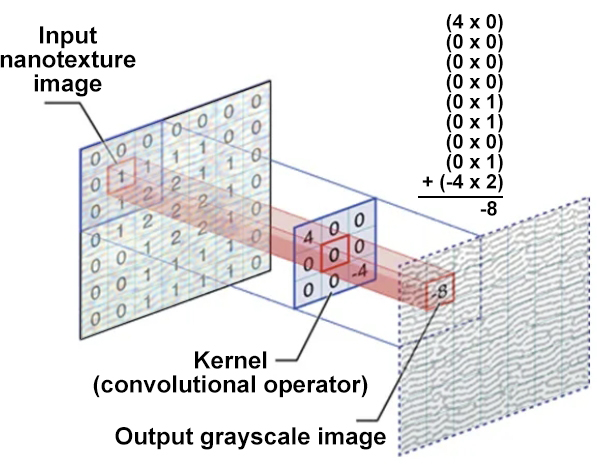


**Figure S11.** Convolution between an input image and a kernel with a size of *k*=3.

In practical applications, the size of the Laplacian operator is typically chosen as an odd number, such as *k*=3, 5, 7, or 9 pixels. This preference for odd-sized kernels is due to the fact that they ensure the center of the operator is exactly at the midpoint of the kernel. This central positioning allows for more precise detection of edge information, as it provides a clear reference point for the convolution operation. In order to more intuitively demonstrate the impact of operator size on edge detection and skeleton analysis, we changed the operator size from *k*=3 to 23, as shown in Figure S12.

**
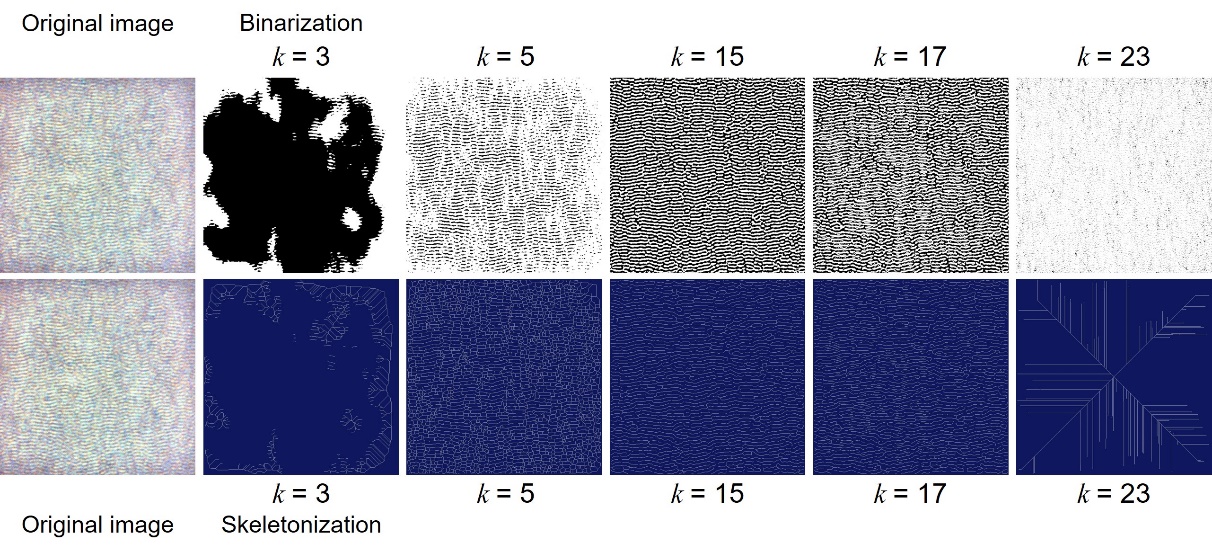
**

**Figure S12.** Edge detection and skeleton analysis of nanotexture with different Laplacian operator sizes.

A smaller operator is more sensitive to fine details, making it suitable for detecting small or intricate nanoripple edges. This sensitivity occurs because smaller operators retain high-frequency information, which is essential for preserving subtle textures or noisy image features. However, smaller operators (e.g. *k*=3 and 5) also amplify noise, which can result in false nanoripple edges if the image contains significant noise. High-frequency noise can interfere with the accurate recognition of textures and cause distortion in the skeletonization process. Conversely, a larger Laplacian operator tends to smooth the image more, making it better for emphasizing prominent, large-scale nanoripple edges while ignoring fine details. This can be advantageous for images with significant noise or indistinct edges, as it reduces the likelihood of false edges. Skeletonization depends on accurate nanoripple edge information, as it works by progressively thinning the nanoripple boundary to form a skeleton. Nevertheless, such improvements have their limits. An operator size that is too large (e.g. *k*=23) can lead to the complete disregard of textures, resulting in the loss of essential features. Therefore, an optimal Laplacian operator capture more detail, which can result in a more connected and accurate skeleton, particularly for objects with complex shapes. This is because a more precise nanoripple edge location aids the skeletonization process in maintaining the structural integrity of the nanotexture.

Similar to edge detection, smaller Laplacian operators are susceptible to noise interference, potentially leading to fragmented skeletons or spurious branches. Larger operators, by smoothing the edges, generally produce a more robust skeletonization output by simplifying the edges, which is especially useful in high-noise scenarios. Selecting the appropriate Laplacian operator size requires a balance based on image characteristics of nanotexture. For images with numerous high-frequency details or for tasks needing precise edge detection, a smaller Laplacian operator is beneficial. In contrast, larger operators yield smoother, more stable results in noisy or detail-insensitive applications. For skeletonization, if the goal is to produce a simplified skeleton, a larger operator can help eliminate unnecessary details and noise. Alternatively, if high skeleton accuracy is needed, it is advisable to use a smaller operator following denoising preprocessing steps to capture finer details of nanotextures.

Estimated encoding capacity of nanotexture with different Laplacian operator sizes is given in Figure S13. The larger the Laplacian operator, the larger length of quantitative unit, which leads to a reduction in encoding capacity. Reducing the Laplacian operator size can significantly enhance encoding capacity, and this improvement is nonlinear. However, as shown in Figure S12, a Laplacian operator that is too small will fail to accurately detect the texture skeleton. Therefore, the selection of the Laplacian operator size should also be optimized based on actual experimental results. After a reasonable range of traversal for the Laplacian operator, we ultimately chose a size of *k*=15 as the Laplacian operator size for skeleton analysis of nanotextures. This choice ensures the accurate detection of nanotextures by computer vision program, while maintaining the encoding capacity of the nanotextures at a reasonable level.


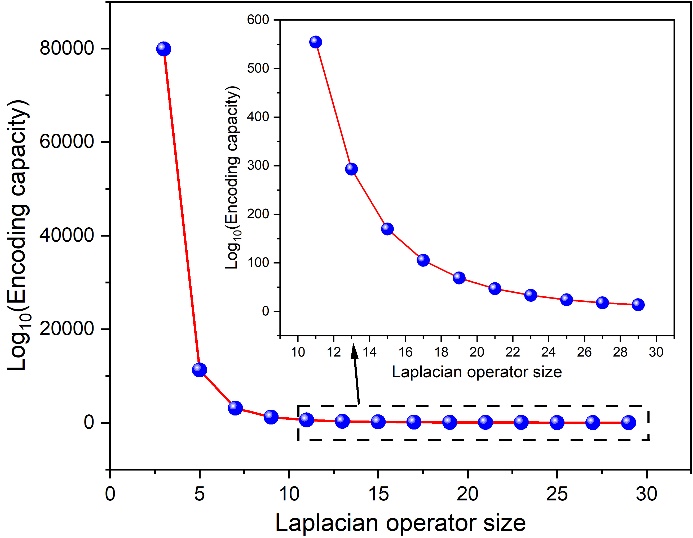


**Figure S13.** Estimated encoding capacity of nanotexture with different Laplacian operator sizes.

The encoding capacity of a single nanotexture block is calculated using a universal encoding model for bitmap PUF as follows^6^.

$\#codes=\left[C(1+L(\frac{1}{\sqrt{D}}-1))^{2}+1\right]^{D\frac{R^{2}}{L^{2}}}$ (S2)

Where *C* is the is the number of colors apart from background of PUF pattern, *L* is the length of quantitative unit, *D* is the effective fill density of PUF pattern, *R* is the readout resolution of PUF pattern. The coding capacity is related to the final decoding method. Taking the example of morphological analysis in our work, the grayscale image of a single nanotexture block is 8-bit binary, the image size is 1024 pixels×1024 pixels, and a Laplacian operator with size of 15 is used to detect the edges of ripples, hence the *L*=15, *R*=1024/15. With the effective fill density of 0.495 based on the average area of the mask, the encoding capacity of a single nanotexture block can be calculated as 10^170^ according to Equation S1, which is exceed the capacity threshold of 10^20^ for unclonable security^12^.

Note S3. Validation workflow of MobileViT.


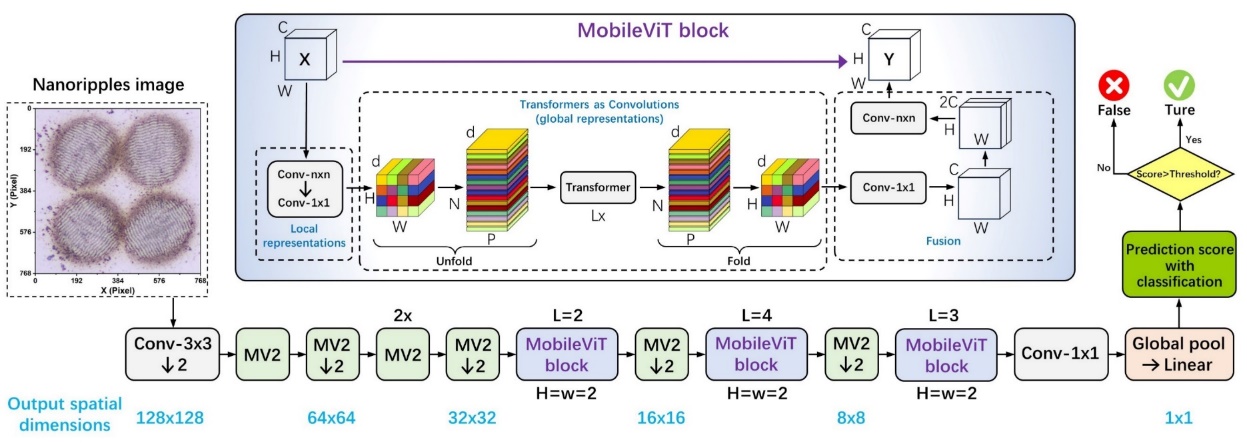


**Figure S14.** Validation workflow of MobileViT.

The validation workflow of MobileViT in our work is depicted in Figure S14. In the workflow scheme, MV2 refers to MobileNetV2 block^13^, blocks that perform down-sampling are marked with 🡫2. MobileViT block: The input X[H, W, C] is shrank by using n×n convolution and PWConv (1×1 convolution) to a channels of d, resulting in X'[H, W, d]. The n×n convolution encodes the local spatial information of X, while the 1×1 convolution is used for dimensionality enhancement (d>C). Then expand X '[H, W, d] to X''[P, N, d], input transformer to extract global spatial information, output Y''[P, N, d], and fold to restore Y'[H, W, d]. Here P=H×W, N=H×W/P. Each patch [P, 1, d] has H×W pixel positions, totaling N patches. Restore Y'[H, W, d] back to [H, W, C] using PWConv, concatenate it with the initial input X[H, W, C] (similar to a shortcut), and use n×n for channel fusion to obtain the final Y.

The nanoripples image to be verified, is performed by a standard 3×3 convolution and 2× down-sampling. Afterwards, it was processed by four MobileNetV2 blocks and subjected to twice 2× down-sampling. The MobileViT block and MobileNetV2 block are added at intervals, using 1×1 convolution for channel compression and global average pooling to output predicted scores with classification labels. Eventually, the authenticity of unidentified document (accuracy of classification), is adjudicated based on a predefined threshold.

Note S4. Augmentation information of tag images.

| Item | Example | Number | Augmentation |
| --- | --- | --- | --- |
| Automatic color enhancement | 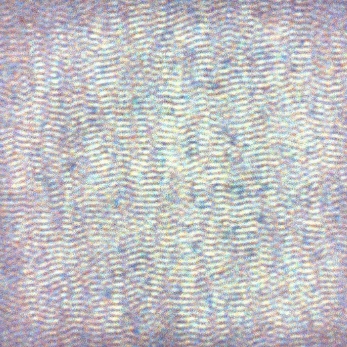 | 1 | Adaptive adjustment of image color balance through ACE algorithm. |
| Rotation | 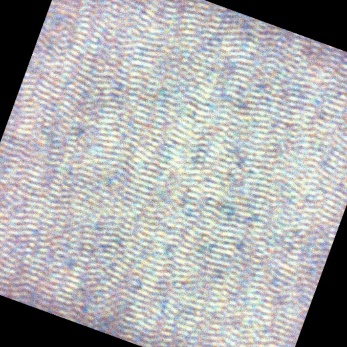 | 17 | Rotate images by 20 to 340 degrees with a step of 20 degrees. |
| Grayscale | 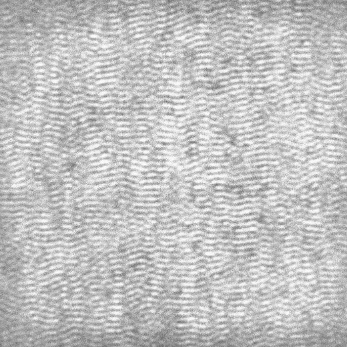 | 5 | Change images to grayscale and overlay them with the original image by varying strengths, effectively removing 20 to 100% of the color. |
| Gamma Contrast | 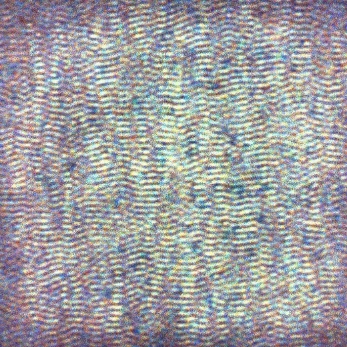 | 5 | Modify the contrast of images by changing Gamma of image. |
| Hue and saturation | 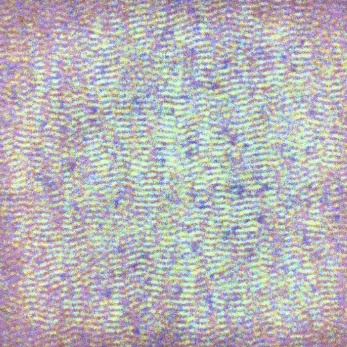 | 6 | Add random values to the hue and saturation, independently per channel and the same value for all pixels within that channel. |
| Color temperature | 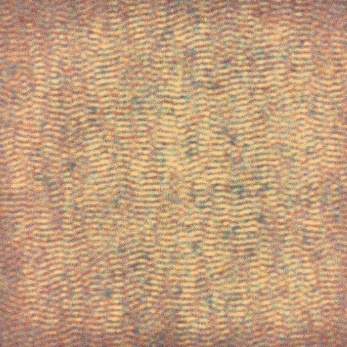 | 7 | Changes the color temperature of images from 2000 and 12000 Kelvin with a step of 2000 Kelvin. |
| Motion blur | 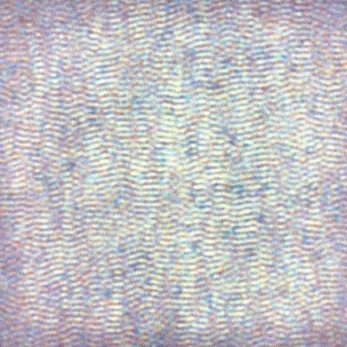 | 3 | Blur images in a way that simulates camera or object movements. |
| Gaussian blur | 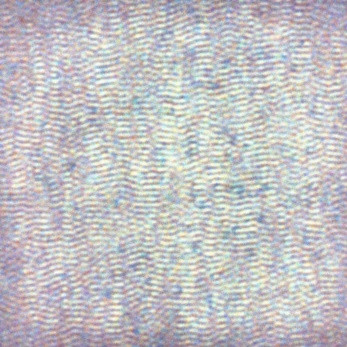 | 2 | Blur each image with a gaussian kernel to simulate defocus. |
| Gaussian noise | 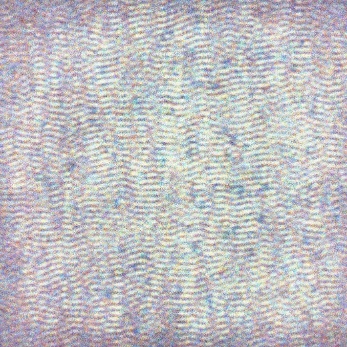 | 3 | Add gaussian noise to an image, sampled once per pixel from a normal distribution *N*(0, *s*), where *s* is sampled per image and varies between 0.1×255 and 0.2×255. |
| Coarse salt noise | 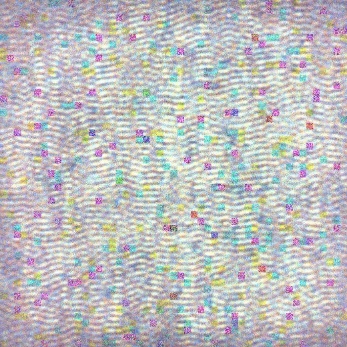 | 4 | Mark 5% of all pixels in a mask to be replaced by salt/pepper noise. The mask has 5% to 20% the size of the input image. |
| Cover | 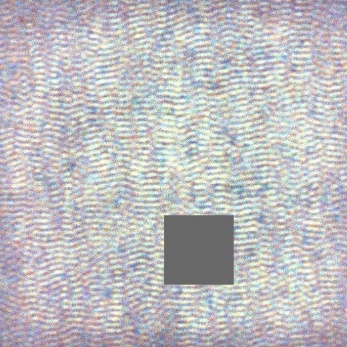 | 5 | Fill 20% of all areas with a random intensity value between 0 and 255, and fill the other 80% of all areas with random colors. |
| Spatters | 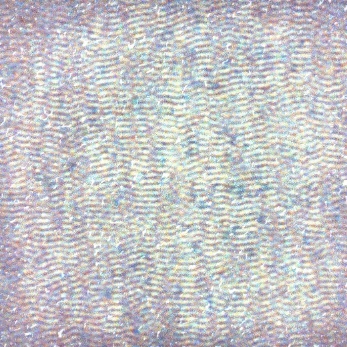 | 2 | Adding different spatters with two severities to simulate pollution. |
| Contrast | 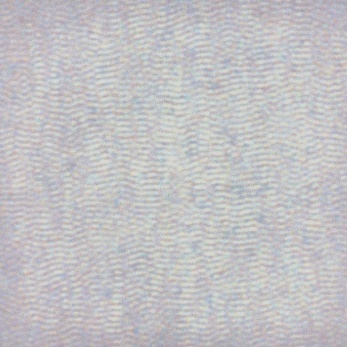 | 2 | Reduce image contrast with two severities to simulate pollution. |
| Brightness | 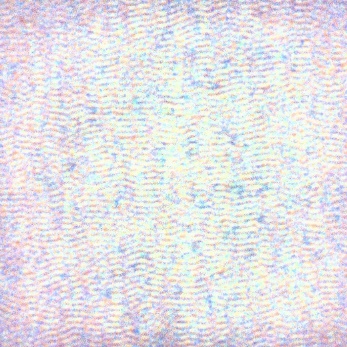 | 2 | Increase image brightness with two severities to simulate overexposure. |
| Total |  | 64 |  |

Video S1. Dynamic structural colors of the PUF micro-QR code. This video demonstrates the variable structural color of the PUF micro-QR code induced by femtosecond laser-induced nanotexture printing.

References

1. Geng J, Feng X, Zhang L, Zhao Z, Yao G, Xu L, et al. Controllable generation of large-scale highly regular gratings on Si films. Light: Advanced Manufacturing 2021, **2**: 22.

2. Geng J, Yan W, Shi L, Qiu M. Surface plasmons interference nanogratings: wafer-scale laser direct structuring in seconds. Light: Science & Applications 2022, **11**: 189.

3. Geng J, Shi L, Liu J, Xu L, Yan W, Qiu M. Laser-induced deep-subwavelength periodic nanostructures with large-scale uniformity. Applied Physics Letters 2023, **122**(2): 021104.

4. Yang J, Feng M, Wang J, Zhao Z, Xu R, Chen Z, et al. Bionic micro-texture duplication and RE3+ space-selective doping of unclonable silica nanocomposites for multilevel encryption and intelligent authentication. Advanced Materials 2023, **35**(49): 2306003.

5. Kim MS, Lee GJ, Leem JW, Choi S, Kim YL, Song YM. Revisiting silk: a lens-free optical physical unclonable function. Nature Communications 2022, **13**(1): 247.

6. Carro-Temboury MR, Arppe R, Vosch T, Sørensen TJ. An optical authentication system based on imaging of excitation-selected lanthanide luminescence. *Science Advances* 2018, **4**(1)**:** e1701384.

7. Bae HJ, Bae S, Park C, Han S, Kim J, Kim LN, et al. Biomimetic microfingerprints for anti-counterfeiting strategies. Advanced Materials 2015, **27**(12): 2083-2089.

8. Kim JH, Jeon S, In JH, Nam S, Jin HM, Han KH, et al. Nanoscale physical unclonable function labels based on block copolymer self-assembly. Nature Electronics 2022, **5**(7): 433-442.

9. Chen G, Weng Y, Wang W, Hong D, Zhou L, Zhou X, et al. Spontaneous formation of random wrinkles by atomic layer infiltration for anticounterfeiting. ACS Applied Materials & Interfaces 2021, **13**(23): 27548-27556.

10. Gu Y, He C, Zhang Y, Lin L, Thackray BD, Ye J. Gap-enhanced Raman tags for physically unclonable anticounterfeiting labels. Nature Communications 2020, **11**(1): 516.

11. Zhang J, Liu Y, Njel C, Ronneberger S, Tarakina NV, Loeffler FF. An all-in-one nanoprinting approach for the synthesis of a nanofilm library for unclonable anti-counterfeiting applications. Nature Nanotechnology 2023, **18**(9): 1027-1035.

12. Arppe R, Sørensen TJ. Physical unclonable functions generated through chemical methods for anti-counterfeiting. *Nature Reviews Chemistry* 2017, **1**(4)**:** 0031.

13. Sandler M, Howard A, Zhu M, Zhmoginov A, Chen L-C. Mobilenetv2: Inverted residuals and linear bottlenecks. Proceedings of the IEEE conference on computer vision and pattern recognition. 2018. p. 4510-4520.
